# Stereo-EEG in Tuberous Sclerosis Complex

**DOI:** 10.15844/pedneurbriefs-34-6

**Published:** 2020-03-03

**Authors:** Jun T. Park, Guadalupe Fernandez-Baca Vaca

**Affiliations:** 1 Department of Neurology, Epilepsy Center, University Hospitals of Cleveland Medical Center, Cleveland, OH; 2 Department of Pediatrics, Division of Neurology & Epilepsy, Rainbow Babies & Children’s Hospital, Cleveland, OH

**Keywords:** Epileptogenic Zone, Stereo-EEG, Tuberous Sclerosis Complex, Pediatric Epilepsy Surgery

## Abstract

A collaborative research team lead by an investigator from the Lyon Neuroscience Research Center and Lyon University Hospital and Lyon 1 University studied epileptogenicity of tuber and its surrounding cortex using stereoelectroencephalography (SEEG) in patients diagnosed with tuberous sclerosis complex (TSC) (genetic or clinical).

A collaborative research team lead by an investigator from the Lyon Neuroscience Research Center and Lyon University Hospital and Lyon 1 University studied epileptogenicity of tuber and its surrounding cortex using stereoelectroencephalography (SEEG) in patients diagnosed with tuberous sclerosis complex (TSC) (genetic or clinical). A study cohort of eighteen patients (11 children) who underwent presurgical SEEG evaluation between 2004 and 2018 was identified from four French tertiary epilepsy centers. The electrodes were implanted bilaterally in 14 patients, and the total number of electrodes ranged from 8 to 16 per patient. The total number of tubers in each patient ranged from 3 to 30 [1].

Epileptogenicity Index (EI) [2] was used to analyze seizures after defining five anatomical regions of interest (ROI): dominant tuber (tuber with highest median EI), perituber cortex, secondary tuber (tuber with second highest median EI), nearby cortex (normal-appearing cortex in the same lobe as the dominant tuber), and distant cortex (normal appearing cortex in other lobes). The value of EI ranged from 0 to 1 (peak epileptogenicity). The epileptogenic zone (EZ) organization was categorized as either focal tuber (EZ limited to dominant tuber with median EI>0.3) or complex (all other patients).

The dominant tuber was the most epileptogenic (P < .001) of the five ROI. Seven patients with a focal tuber EZ organization had 80% Engel IA postsurgical outcome and the following 4 tuber characteristics: continuous interictal discharges (100%), fluid-attenuated inversion recovery (FLAIR) hypointense center (86%), stimulation – induced seizures (71%), and center-to-rim EI gradient. A combination of the first three characteristics showed a 98% specificity for a focal tuber EZ organization. Six patients with a complex EZ organization showed 40% Engel IA outcome with nearby cortex (4 patients) and distant cortex (1) as the most epileptogenic region. The authors concluded that tubers with focal EZ organization are much like type II focal cortical dysplasia, and that identification of these tubers relate to EZ hypothesis generation and invasive EEG/resection strategies. [1]

COMMENTARY. Talarirach and Bancaud developed “stereoencephalographie” in the 1960s as a tool to aid in defining the EZ. Their definition of EZ was an ictal electro-clinical concept based on SEEG recordings. Over the past 50 years, the concept of EZ has evolved due to invention of sophisticated localizing tools. Hans Lüders et al. (2006) defined EZ as “the minimum amount of cortex that must be resected to produce seizure freedom [3].” Ultimately, identification of the EZ heavily influences seizure freedom after resective surgery. This article adds an important insight for identification of EZ in TSC, utilizing one of the advantages of SEEG that allows sampling of multiple cortical regions, unilateral and/or bilateral.

The authors reported the following results in consideration of EZ identification in TSC: 1. A combination of the three characteristics - continuous interictal discharges, FLAIR hypointense center, and stimulation-induced seizures, showed a 98% specificity in localizing the dominant tuber; 2. Nearby cortex (4 of 6 patients) was the most epileptogenic (with highest medial EI) in the presence of a dominant tuber that lacked center-to-rim gradient in EI. Importantly, these results may contribute to development of an effective implantation strategy of intracerebral electrodes in further defining EZ in TSC, as one of the challenges of using SEEG is determining targets and trajectories based on presurgical data and EZ hypothesis. Experience and skill of a functional neurosurgeon who places intracerebral electrodes to confirm/modify/reject the hypothesis of EZ may also influence outcome. The current work is a welcome and laudable contribution to further our understanding of epileptogenicity in TSC.

## Disclosures

The authors have declared that no competing interests exist.
